# Annual of the Universal Medical Sciences

**Published:** 1890-01

**Authors:** 


ANNUAL OF THE UNIVERSAL MEDICAL SCIENCES, A YEARLY REPORT OF THE PROGRESS OF THE GENERAL SANITARY SCIENCES THROUGHOUT THE WORLD. Edited by Charles E. Sajons, M. D., and seventy associated editors, assisted by over two hundred corresponding editors, etc. Illustrated 1889, F. A. Davis, publisher, New York, Philadelphia and London.

The second issue of this series is now before the profession and presents many improvements over its excellent predecessor.

Among its new features are a chapter on Life Insurance and a double reduction of weights and measures. Also the reduction of both Centigrade and Reaumur readings, to Fahrenheit, and *vice versa*, which is of very great convenience to the general practitioner.

Of the series as a whole too much cannot be said in praise, though there are a few chapters that do not come up to the general high standard.

As a work of reference it is invaluable to all classes of physicians, presenting as it does the gist of the best current medical literature throughout the world. The admirably arranged references enable the reader to follow up any subject which he may desire to investigate more thoroughly.

The work abundantly deserves the continued liberal patronage of the profession. F. W. M.

